# Extracorporeal Membrane Oxygenation for SARS-CoV-2 Acute Respiratory Distress Syndrome: A Retrospective Study From Hubei, China

**DOI:** 10.3389/fmed.2020.611460

**Published:** 2021-01-12

**Authors:** Xiaobo Yang, Ming Hu, Yuan Yu, Xijing Zhang, Minghao Fang, Yingtao Lian, Yong Peng, Lingling Wu, Yongran Wu, Jun Yi, Lu Zhang, Bing Wang, Zhengqin Xu, Boyi Liu, Yadong Yang, Xiaowei Xiang, Xingguang Qu, Wenhao Xu, Hunian Li, Zubo Shen, Changming Yang, Fengsheng Cao, Jie Liu, Zhaohui Zhang, Lianghai Li, Xiaoyun Liu, Ruiting Li, Xiaojing Zou, Huaqing Shu, Yaqi Ouyang, Dan Xu, Jiqian Xu, Jiancheng Zhang, Hong Liu, Hong Qi, Xuepeng Fan, Chaolin Huang, Zhui Yu, Shiying Yuan, Dingyu Zhang, You Shang

**Affiliations:** ^1^Department of Critical Care Medicine, Union Hospital, Tongji Medical College, Huazhong University of Science and Technology, Wuhan, China; ^2^Department of Critical Care Medicine, Wuhan Pulmonary Hospital, Wuhan, China; ^3^ICU Center of Xijing Hospital, Airforce Medical University, Xi'an, China; ^4^ICU of Huoshenshan Hospital, Wuhan, China; ^5^Department of Critical Care Medicine, Tongji Hospital, Tongji Medical College, Huazhong University of Science and Technology, Wuhan, China; ^6^Department of Critical Care Medicine, Renmin Hospital of Wuhan University, Wuhan, China; ^7^Intensive Care Unit, Xiehe Wuhan Red Cross Hospital, Wuhan, China; ^8^Department of Cardiothoracic Surgery, The First People's Hospital of Jingmeng, Jingmeng, China; ^9^Department of Critical Care Medicine, Xiangyang Central Hospital, Affiliated Hospital of Hubei University of Arts and Science, Xiangyang, China; ^10^Department of Critical Care Medicine, Jingzhou Central Hospital, The Second Clinical Medical College, Yangtze University, Jingzhou, China; ^11^Department of Critical Care Medicine, Xiangyang No.1 People's Hospital, Affiliated Hospital of Hubei University of Medicine, Xiangyang, China; ^12^Department of Critical Care Medicine, Taihe Hospitai Affiliated to Hubei University Medicine, Shiyan, China; ^13^Department of Critical Care Medicine, Huanggang Central Hospital, Huanggang, China; ^14^Department of Critical Care Medicine, Dongfeng Hospital, Affiliated to Hubei University of Medicine, Shiyan, China; ^15^Department of Intensive Care Unit, The First College of Clinical Medical Sciences, China Three Gorges University, Yichang, China; ^16^Department of Critical Care Medicine, Xiaogan Central Hospital, Xiaogan, China; ^17^Emergency and Critical Care Center, Renmin Hospital, Hubei University of Medicine, Shiyan, China; ^18^Department of Critical Care Medicine, Ezhou Central Hospital, Ezhou, China; ^19^Department of Anesthesiology, The First People's Hospital of Jingmeng, Jingmen, China; ^20^Department of Critical Care Medicine, Wuhan No.1 Hospital, Wuhan, China; ^21^Research Center for Translational Medicine, Jinyintan Hospital, Wuhan, China

**Keywords:** SARS-CoV-2, COVID-19, acute respiratory distress syndrome, extracorporeal membrane oxygenation, intensive care unit, prognosis

## Abstract

**Background:** The data on long-term outcomes of patients infected by SARS-CoV-2 and treated with extracorporeal membrane oxygenation (ECMO) in China are merely available.

**Methods:** A retrospective study included 73 patients infected by SARS-CoV-2 and treated with ECMO in 21 intensive care units in Hubei, China. Data on demographic information, clinical features, laboratory tests, ECMO durations, complications, and living status were collected.

**Results:** The 73 ECMO-treated patients had a median age of 62 (range 33–78) years and 42 (63.6%) were males. Before ECMO initiation, patients had severe respiratory failure on mechanical ventilation with a median PO_2_/FiO_2_ of 71.9 [interquartile range (IQR), 58.6–87.0] mmHg and a median PCO_2_ of 62 [IQR, 43–84] mmHg on arterial blood analyses. The median duration from symptom onset to invasive mechanical ventilation, and to ECMO initiation was19 [IQR, 15–25] days, and 23 [IQR, 19–31] days. Before and after ECMO initiation, the proportions of patients receiving prone position ventilation were 58.9 and 69.9%, respectively. The median duration of ECMO support was 18.5 [IQR 12–30] days. During the treatments with ECMO, major hemorrhages occurred in 31 (42.5%) patients, and oxygenators were replaced in 21 (28.8%) patients. Since ECMO initiation, the 30-day mortality and 60-day mortality were 63.0 and 80.8%, respectively.

**Conclusions:** In Hubei, China, the ECMO-treated patients infected by SARS-CoV-2 were of a broad age range and with severe hypoxemia. The durations of ECMO support, accompanied with increased complications, were relatively long. The long-term mortality in these patients was considerably high.

## Background

In late December of 2019, a new highly transmittable coronavirus struck Wuhan City, Hubei Province, China, the first known epicenter by far (1, 2). Very quickly, the genome sequence was identified, which shares 79% genomic sequence identity to SARS-CoV (3). The coronavirus was named SARS-CoV-2 by the Coronavirus Study Group of the International Committee on Taxonomy of Viruses (4). The virus causes a spectrum of diseases, named coronavirus disease 2019 (COVID-19) by WHO on February 11th, 2020 (5). The Chinese government took aggressive measures, including the lockdown of all cities in Hubei Province, and succeeded in stopping the spread of the virus in mainland China (6, 7).

Although when and where the spread of SARS-CoV-2 began at the very beginning and how it turned into a worldwide pandemic have yet to determine (8–10), the fact that some patients infected by SARS-CoV-2 developed severe acute respiratory distress syndrome (ARDS) is indisputable (11, 12). Based on the WHO interim guidance, extracorporeal membrane oxygenation (ECMO) was recommended to treat patients with refractory hypoxemia, hypercapnia, or both (13). Recently, Barbaro et al. reported ECMO support in COVID-19 based on an online registry from the Extracorporeal Life Support Organization (ELSO) with only 52 cases from Asia Pacific area (14). Chinese critical care physicians have been utilizing since the beginning of SARS-CoV-2 epidemic in Wuhan City, and later in other cities of Hubei Province. However, the characteristics, severity of respiratory failure, duration of ECMO, complications, and long-term outcomes of patients with severe ARDS caused with SARS-CoV-2 treated with ECMO in Hubei, China are far beyond knowledge (15).

## Methods

### Study Design and Patient Eligibility

We conducted this retrospective observational study on adult patients infected by SARS-CoV-2 who were treated with ECMO since January 1 in Hubei, China. Twenty-one intensive care units (ICUs) that provided ECMO support to adult ARDS patients with COVID-19 during the study period contributed to this study. The infection of SARS-CoV-2 was confirmed in all included patients based on the WHO interim guidance and the guidance of National Health Commission of the People's Republic of China (13, 16). ARDS was defined according to the guidance of WHO for COVID-19 (13). We excluded patients reported in previous studies (11, 12, 15). The initiation of ECMO was guided by WHO recommendations (13) and at the discretion of treating physicians.

### Data Collection

De-identified data were collected by the ICU director or designated physicians using a care form. A web conference was held, if necessary, to help data collectors in each ICU. We collected data on age, sex, location, occupation, medical histories, the date of symptom onset, invasive mechanical ventilation and ECMO initiation, laboratory tests at and before ECMO initiation, treatments before and after ECMO initiation (renal replacement therapy, prone position ventilation, steroid therapy, convalescent plasma), type of mechanical ventilator, its settings and monitoring data right before and after ECMO initiation (tidal volume, respiratory rate, positive end-expiratory pressure, minute volume), ECMO mode and its settings in the day after ECMO initiation (gas flow and pump flow), duration of ECMO, complications directly related to ECMO (hemorrhage, oxygenator replacement and cannula replacement) and outcomes by May 31, 2020. Hemorrhagic complications were categorized into major and minor hemorrhage and the former was defined as cerebral hemorrhage or other organ hemorrhage that necessitated reduction or cessation of infusing anticoagulating drugs for at least 6 h and/or other intervention, including endoscopic hemostasis or interventional arterial embolization.

### Statistical Analysis

Due to the exploratory nature of this study, we included eligible patients as many as possible. Data were expressed as median [interquartile range (IQR)] or median [range] for continuous variables, and count (%) for categorical variables. Due to the small number of ECMO-treated patients who were alive, no comparison was conducted between the survivors and non-survivors. Kaplan–Meier method was used to depict the probability of survival since the day of ECMO initiation. Log-rank test was used to compare survival data. A two-sided *p* < 0.05 were considered statistically significant. The Stata/IC 15.1 software (StataCorp, College Station, TX, USA) was used for all analyses.

## Results

**Figure 1:**
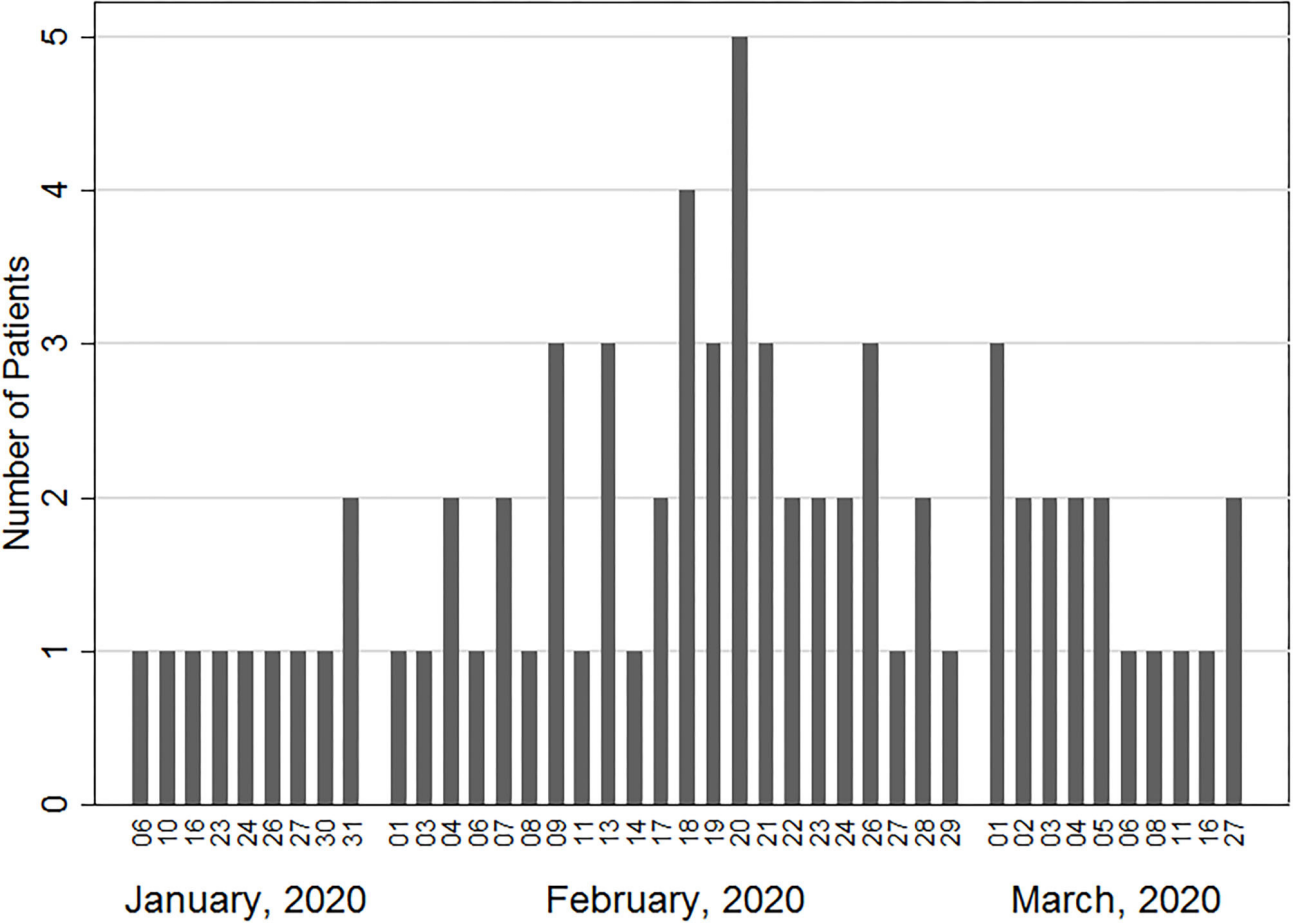
Number of ECMO-treated patients with COVID-19 in association with the date of ECMO initiation.

Seventy-three ARDS patients from 21 ICUs in Hubei, China were included (Figure 1). The patients had a median age of 62 [range 33–78] years, 23 (31.5%) were aged ≥ 65 years, and 46 (63.0%) were males (Table 1). 53 (72.6%) were treated in Wuhan City. As for preexisting comorbidities, 10 (13.7%), 27 (37.0%), 13 (17.8%), and 5 (6.9%) patients had coronary artery disease, hypertension, diabetes, and chronic obstructive pulmonary disease, respectively. None had chronic liver disease, cerebral vascular disease, connective tissue disease, malnutrition, or dementia.

**Table 1:** Characteristics of COVID-19 patients treated with ECMO.

**Characteristics**	**ECMO (*n* = 73)**
Age, years	62 [51–66]
≥ 65 years	23 (31.5%)
Male	46 (63.0%)
APACHE II	19 [16–21]
Preexisting comorbidities	
Coronary artery disease	10 (13.7%)
Hypertension	27 (37.0%)
Diabetes	13 (17.8%)
Chronic obstructive pulmonary disease	5 (6.8%)
Malignancy	1 (1.4%)
Smoking	4 (5.5%)

*Data are expressed as median [interquartile range] or count (%) unless specified*.

*COVID-19, coronavirus disease 2019; ECMO, extracorporeal membrane oxygenation; IMV, invasive mechanical ventilation; APACHE II, Acute Physiology and Chronic Health Evaluation II*.

For all the patients, severe hypoxemia [PO_2_/FiO_2_, median (IQR), 71.9 (58.6–87.0) mmHg] and hypercapnia [PCO_2_, median (IQR), 62 (43–84) mmHg] were identified. Based on their outcomes, the results of laboratory tests of ECMO treated patients on the day of ECMO initiation were presented separately in Table 2.

**Table 2:** Laboratory tests on the day of ECMO initiation in 73 patients with COVID-19.

**Characteristics**	**Non-survivors (*n* = 59)**	**Survivors (*n* = 14)**
Hemoglobin, g/l	103 [88–118]	108 [95–118]
White blood cell, × 10^9^/l	15.75 [11.91–20.55]	9.53 [6.29–12.72]
Neutrophils, × 10^9^/l	14.23 [10.20–19.15]	8.27 [5.58–11.9]
Lymphocytes, × 10^9^/l	0.47 [0.33–0.80]	0.60 [0.47–0.67]
Platelet, × 10^9^/l	150 [92–184]	179 [78–223]
Alanine transaminase, U/l	23 [20–41]	45 [34–84]
Aspartate transaminase, U/l	33 [23–52]	46 [24–97]
Total bilirubin, mmol/l	13.7 [7.9–25.9]	17.5 [13.7–29.3]
Direct bilirubin, mmol/l	6.3 [4.0–12.7]	10.0 [4.4–14.8]
Albumin, g/l	31.6 [28.2–35.0]	35.6 [30.4–37.6]
Sodium, mmol/l	142 [140–145]	143 [140–147]
Potassium, mmol/l	3.94 [3.64–4.84]	4.03 [3.76–4.64]
Creatinine, umol/l	70.0 [51.0–109.0]	78.0 [60.0–84.0]
Blood urea nitrogen, mmol/l	10.02 [7.32–14.05]	8.20 [6.79–11.27]
Prothrombin time, s	14.8 [12.5–17.1]	15.1 [13.9–16.3]
Activated partial thromboplastin time, s	32.4 [26.4–40.0]	34.6 [30.1–40.4]
Fibrinogen, g/l	3.94 [2.39–4.79]	4.31 [2.53–6.25]
D-dimer, mg/l	7.36 [4.35–19.34]	6.34 [3.66–11.36]
Arterial blood gas analysis		
pH	7.35 [7.27–7.43]	7.31 [7.24–7.41]
PO_2_, mmHg	64.5 [55.0–78.0]	70.0 [54.0–72.0]
PCO_2_, mmHg	60.2 [43.0–80.0]	63.9 [50.0–85.0]
PO_2_/FiO_2_, mmHg	71.6 [57.0–87.0]	72.0 [60.0–85.8]
HCO_3_-, mmol/l	30.0 [25.7–34.1]	32.1 [22.8–34.8]
Lactate, mmol/l	2.1 [1.6–3.1]	2.2 [1.2–2.8]

*Data are expressed as median [interquartile range]*.

*COVID-19, coronavirus disease 2019; ECMO, extracorporeal membrane oxygenation; PO_2_, partial pressure of oxygen; PCO2, partial pressure of carbon dioxide; FiO_2_, fraction of inspired oxygen*.

The median duration from symptom onset to invasive mechanical ventilation, and to ECMO initiation was 19 [IQR, 15–25] days, and 23 [IQR, 19–31] days, with details listed in Table 3 separately based on the outcomes of the patients. Only 1 (1.4%) patient had ECMO initiated while receiving non-invasive mechanical ventilation. Two days later, the patient was intubated and ventilated invasively. All other patients were deeply sedated and/or paralyzed while being cannulated. The mode was veno-venous ECMO in all patients, with a median pump flow of 3.5 [IQR 3.2–4.0] L/min and an oxygen flow of 4.5 [IQR, 4.0–5.0] L/min. Before ECMO, pneumothorax and thrombocytopenia occurred in 1 (1.4%) patient and 26 (36.1%) patients, and after ECMO initiation, they occurred in 10 (13.7%) more patients and 33 (45.2%) more patients, respectively. Before ECMO, 14 (19.2%) patients were diagnosed with hospital acquired infection, and after ECMO initiation, 42 (57.5%) patients were diagnosed with it. After ECMO initiation, the proportion of patients receiving renal replacement therapy, prone position ventilation and convalescent plasma increased from 20.5, 58.9, and 12.3% to 71.2, 69.9, and 31.5%, respectively.

**Table 3:** Treatments and mechanical ventilation before, at and after ECMO initiation in patients with COVID-19.

**Characteristics**	**Non-survivors (*n* = 59)**	**Survivors (*n* = 14)**
**Before ECMO initiation**		
Confirmed hospital acquired infection	11 (18.6%)	3 (21.4%)
Renal replacement therapy	14 (23.7%)	1 (7.1%)
Prone position ventilation	39 (66.1%)	4 (28.6%)
Steroid therapy	51 (86.4%)	11 (78.6%)
Convalescent plasma	7 (11.8%)	2 (14.3%)
**At ECMO initiation**		
Duration		
From symptom onset to hospitalization, days	6 [3–9]	6.5 [3–12]
From symptom onset to IMV initiation, days	19 [14–25]	20 [17–29]
From symptom onset to ECMO initiation, days	23 [18–32]	24 [19–29]
From IMV initiation to ECMO initiation, days	4 [1–7]	1.5 [0–6]
Concomitant mechanical ventilation		
IMV	58 (98.3%)	14 (100.0%)
NIV	1 (1.7%)	0 (0.0%)
Ventilator setting and monitoring before ECMO initiation		
Tidal volume, ml	400 [350–410]	400 [360–440]
Respiratory rate, breaths/minute	25 [20–28]	23 [18–26]
PEEP, cmH2O	10 [8–12]	10 [8–12]
Minute volume (n = 43), L/min	9.3 [8.1–10.2]	8.1 [6.5–10.8]
**In the day later after ECMO initiation**		
Veno-venous ECMO		
ECMO setting		
Pump Flow, L/min	3.5 [3.2–4.0]	3.3 [3.1–3.5]
Oxygen flow, L/min	4.2 [4.0–5.0]	4.5 [3.0–5.0]
Ventilator setting and monitoring after ECMO initiation		
Tidal volume, ml	300 [240–360]	260 [165–380]
Respiratory rate, breaths/minute	14 [10–20]	12 [10−15]
PEEP, cmH2O	10 [0]	10 [6–10]
Minute volume, L/min	4.3 [3.1–6.5]	3.9 [2.1–4.9]
**After ECMO initiation, by May 31, 2020**		
Confirmed hospital acquired infection	34 (57.6%)	8 (57.1%)
Renal replacement therapy	43 (72.9%)	9 (64.3%)
Prone position ventilation	40 (67.8%)	11 (78.6%)
Steroid therapy	38 (64.4%)	7 (50.0%)
Convalescent plasma	16 (27.1%)	7 (50.0%)

*Data are expressed as count (%) or median [interquartile range]*.

*ECMO, extracorporeal membrane oxygenation; COVID-19, coronavirus disease 2019; IMV, invasive mechanical ventilation; NIV, noninvasive mechanical ventilation; PEEP, positive end-expiratory pressure*.

Major hemorrhage occurred in 31 (42.5%) patients, with details listed in Table 4 separately based on the outcomes of the patients. Major gastrointestinal hemorrhage occurred in 25 (34.3%) patients, with 3 (4.1%) having concurrent cerebral hemorrhage and 4 (5.5%) having respiratory tract hemorrhage. Another 2 (2.7%) only had cerebral hemorrhage, and another 2 (2.7%) only had and respiratory tract hemorrhage. In 1 (1.5%) patient, massive bladder hemorrhage was treated with endoscopic interventions and subsequent arterial embolization. In another patient, massive splenic hemorrhage was also treated with arterial embolization. The most common minor hemorrhage was ECMO cannula associated hemorrhage, which occurred in 24 (32.9%) patients. The mode was switched to veno-arterial ECMO in one patient and to veno-veno-arterial ECMO in two patients, and all the three patients deceased before weaning off ECMO. During the treatments, oxygenators were replaced in 21 (28.8%) patients, with cannulae replaced concurrently in 7 (9.6%) patients. Among all patients included, 21.5 [IQR, 11–45.5] units, 4 [IQR, 1–8] units, 2,200 [IQR, 600–5,500] milliliters, and 4.25 [IQR, 0–20] units of red blood cell, platelet, plasma, and cryoprecipitate were transfused during the treatment with ECMO, respectively. The analgesics, sedatives, and paralytics used during the treatment with ECMO were presented in Supplementary Table 1.

**Table 4:** Complications of ECMO, and blood transfusion in 73 patients with COVID-19.

**Characteristics**	**Non-survivors (*n* = 59)**	**Survivors (*n* = 14)**
**Complications**		
Major hemorrhage		
Cerebral hemorrhage	5 (8.5%)	0 (0.0%)
Gastrointestinal hemorrhage	20 (33.9%)	5 (35.7%)
Respiratory tract hemorrhage	6 (10.1%)	0 (0.0%)
Bladder hemorrhage	0 (0.0%)	1 (7.1%)
Splenic hemorrhage	1 (1.7%)	0 (0.0%)
Minor hemorrhage		
Cannula associated hemorrhage	15 (25.4%)	9 (64.3%)
Chest tube associated hemorrhage	4 (6.8%)	0 (0.0%)
Gastrointestinal hemorrhage	4 (6.8%)	0 (0.0%)
Nasal cavity hemorrhage	0 (0.0%)	1 (%)
ECMO adjustment		
Switch mode^*^	3 (5.1%)	0 (0.0%)
Oxygenator replacement	15 (25.4%)	6 (42.9%)
Cannula replacement	4 (6.8%)	3 (21.4%)
**Blood transfusion**		
Red blood cell, Units	19.5 [10–42]	41 [22–53.5]
Platelet, Units	4 [1–8]	4.5 [1–8]
Plasma, milliliters	1500 [600–4400]	5350 [1250–8400]
Cryoprecipitate, Units	4.25 [0–18]	2.5 [0–66.5]
Albumin, grams	295 [160–550]	465 [280–770]

*Data are expressed as count (%) or median [interquartile range]*.

*ECMO, extracorporeal membrane oxygenation; COVID-19, coronavirus disease 2019; ICU, intensive care unit*.

* *One patient to veno-arterial ECMO and another two patients to veno-veno-arterial ECMO*.

**Figure 2:**
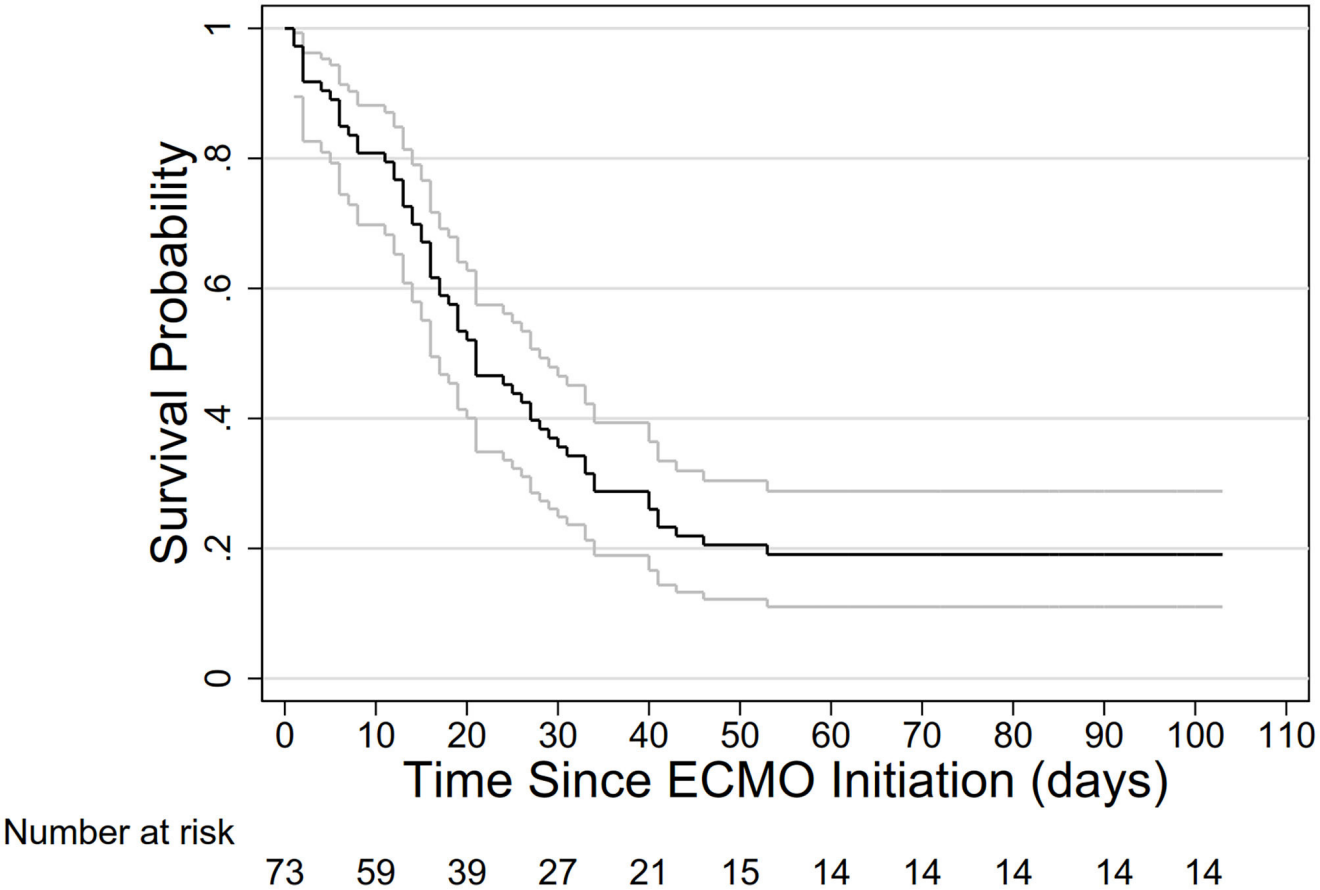
Survival probability in 73 ECMO-treated patients with ARDS caused by SARS-CoV-2. ECMO, extracorporeal membrane oxygenation; ARDS, acute respiratory syndrome.

Since ECMO initiation, 46 (63.0%) deceased by 30 days, and 59 (80.3%) by 60 days (Figure 2), comprising 53 deceased before decannulation and 6 after decannulation. By May 31, 2020, 4 (5.5%) patients were still in ICUs on invasive ventilator, comprising of 2 (2.7%) on and another 2 (2.7%) off ECMO. For the two patients still on ECMO, one had been on ECMO for 65 days, and the other for 95 days. 7 (9.6%) patients were discharged home, and 3 (4.1%) were transferred to general wards. The median duration of ECMO therapy was 17 [IQR 11–29] days, with no significant difference between those successfully and unsuccessfully weaned off ECMO [median (IQR), 17.5 (13–35) in 18 patients and 17 (8–27) in 53 patients].

## Discussion

This multicenter study on the use of ECMO for patients with ARDS caused by SARS-CoV-2 showed that patients of a broad age range were treated with ECMO in Hubei, China, for a relatively long duration and the mortality of these patients were considerably high.

To our best knowledge, this is the first study with long-term follow-ups on more than 70 ECMO-treated patients infected by SARS-CoV-2 in China. Before our study, we identified only three small sample-sized case series of Chinese patients with COVID-19 treated with ECMO. In a single-centered report of eight patents from Shanghai, China, four patients deceased with a mean duration of 30.5 days since ECMO initiation (17). In another study from 2 ICUs from Hubei, China, only 12 ECMO-treated COVID-19 patients with a mean follow-up period of 11.3 days were included, and none deceased (18). Their mean was, and all these patients were not included in ours study.

Yang et al. included 21 ECMO-treated patients infected by SARS-CoV-2 from Hubei, China, and they found that ECMO-treated patients had a mortality of 57.1%, which was not significantly different from that of patients treated with IMV only (15). With none of the patients from the last two studies included, we did not find significant difference on mortality between ECMO-treated COVID-19 patients and COVID-19 patients treated with IMV only (80.8 vs. 71.2%). The mortality of ECMO-treated patients in our study was higher than in the study of Yang et al. (15), which is most likely because of the longer durations of follow-up and the higher scores of Acute Physiology and Chronic Health Evaluation II.

Outside China, two major studies on ECMO support in COVID-19 patients were identified. In a cohort study of 83 ECMO-treated COVID-19 patients from five ICUs of one university hospital network, Schmidt et al. estimated that 31% of patients deceased at 60 days (19). The mortality of a longer term was unknown, because 24% of patients were still in ICUs in the study of Schmidt et al. (19) and Shekar et al. (20). Based on ELSO Registry of ECMO in COVID-19 with 779 ECMO-treated patients with ARDS, Barbaro et al. found that their median age was 50 years and estimated that their in-hospital mortality at 90-day was 38.0% (95% confidence interval 34.6–41.5%) (14). However, it rose up to 43% at the end of October, 2020 (21).

The considerably high mortality in ECMO-treated patients with ARDS was mainly the result of high mortality of critically ill patients infected by SARS-CoV-2. In our previous study, we found that 61.5% critically ill patients deceased by 28 days after ICU admission (12). In the first series of critically ill patients admitted to the ICU between February 20, 2020, and March 5, 2020 at Evergreen Hospital, Washington, USA, by March 17, 2020, the mortality was 67% (22). In a study of 1,591 critically ill patients from Lombardy Region, Italy, 26% died in ICUs and 58% were still in ICU for a median follow-up of 9 days (23). In 2,626 patients who were either discharged or deceased with a hospitalization day of 4.1 [IQR 2.3–6.8] days, 282 (88.1%) of 320 patients who received mechanical ventilation deceased (24), which clearly indicated that critically ill patients were at great risk of death. The fundamental basis for high risk of death in critically patients infected by SARS-CoV-2 is due to the lack of specialized drug or therapy (25). However, another crucial aspect was that except the modest sample-sized study from Washington, USA (22), all the three studies were from the most severely struck places, where medical resources were overwhelmed (12, 23, 24). It was reasonable that as a subgroup of the most critically ill patients, ECMO-treated patients with ARDS would definitely follow the same path.

Another factor contributing to the high mortality in our cohort was the median age was relatively high, with 31.8% patients being older than 65 years. Determining who should receive ECMO is even more challenging, which entangles medical, technical, financial and ethical considerations (26). In face of the fact that about 5% of patients infected by SARS-CoV-2 are critically ill patients and also possible candidates for ECMO, the depletion of medical resources makes the situation much more difficult, especially in area struck hardest by the pandemic (15, 27, 28). Our government deployed more than 40,000 health care workers from other provinces to Hubei province to contain the outbreak. By mid-February, 2020, one third of health care workers specialized in critical care medicine in China were treating COVID-19 patients in Hubei (29). ECMO machines, were also transported to Hubei at the same time (30). And all expenses were covered by our government. The rapid mobilization of medical resources made it possible to put more patients older than 65 years on ECMO.

There were other findings worth noting when treating COVID patients with ECMO. First, the duration of ECMO in COVID-19 may be relatively longer. The median duration of ECMO support in the study of Schmidt et al. was 20 days (19), and it was 18.5 days in our study, both of which were longer than a median of 13.9 days reported by Barbaro et al. (14) and a median duration of 10 days in ECMO-treated patients with 2009 influenza A (31, 32). Second, major hemorrhage often occurred. Almost the same to our finding, Schmidt et al. reported that 42% patients had major hemorrhages, defined as severe hemorrhagic events, intracerebral hemorrhages, or other hemorrhages causing a fatal outcome (19). Generally, unfractionated heparin was infused continuously to achieve an activated partial thromboplastin time (APTT) of 60–80 s in Hubei, China. An APTT of 60–75 s or an anti-Xa activity of 0·3–0·5 IU/mL was targeted for anticoagulation by Schmidt et al. (19). Third, oxygenator failure also often occurred. In comparison to an oxygenator replacement rate of 28.8% in our study, circuit change, oxygenator failure, pump failure or cannula problems occurred in 28% patients from the ELSO Registry of ECMO in COVID-19 (14). For patients without COVID-19, oxygenator failure occurred in only 6.6% of adult ECMO-treated patients, according to Extracorporeal Life Support Organization Registry International Report 2016 (33). Fourth, determining the appropriate time of ECMO initiation is challenging. In 2009 H1N1 pandemic, the PaO_2_/FiO_2_ was 56 [IQR, 48–63], while 20% of patients received prone position ventilation before ECMO initiation and the mortality was 21% (31). The PaO_2_/FiO_2_ value was similar to that in our study, but even with a considerable increase in the proportion of patient receiving prone position ventilation, the mortality was almost quadrupled. An earlier ECMO initiation seems necessary (34). Fifth, the settings of mechanical ventilators after ECMO initiation, especially tidal volume and respiratory rate, may vary between studies. A median tidal volume of 2.5 ml/kg predicted body weight, much lower than that of our study and a median respiratory rate of 20 breaths/min, much higher than that of our study, were set in COVID-19 patients treated by Schmidt et al. (19) However, both settings of mechanical ventilator after ECMO initiation in our study were close to these from another study of Schmidt et al. In a prospective study covering data from 350 ECMO-treated patients with ARDS in 23 ICUs, Schmidt et al. reported that the tidal volume was 3.7 ± 2.0 ml/kg and respiratory rate was 14 ± 6 breaths/min when patients were on ECMO (35).

This study has some limitations. First, this is a retrospective study. Some critical information, such as ventilator settings, was incomplete. The data on demographic information, complications, timing of critical events, and living status, however, are concrete. Second, the shortage of health care workers in Hubei Province, especially in its capital–Wuhan City, may make some findings in our study difficult to interpret. However, a shortage of health care workers is or will be a common reality in many places right now or in the near future.

## Conclusions

The ECMO-treated patients with ARDS caused by SARS-CoV-2 were of a broad age range and with severe hypoxemia in Hubei, China. The duration of ECMO support was relatively long, and the rate of complications and the mortality were high.

## Data Availability Statement

The original contributions presented in the study are included in the article/Supplementary Materials, and further inquiries can be directed to the corresponding author/s.

## Ethics Statement

The studies involving human participants were reviewed and approved by the Ethics Committee of Union hospital, Tongji Medical College, Huazhong University of Science and Technology (2020-0103-1). Written informed consent for participation was not required for this study in accordance with the national legislation and the institutional requirements.

## Author Contributions

XY, MH, YYu, XZh, MF, YL, YP, LW, YW, JY, LZ, BW, ZX, BL, YYa, XQ, WX, HL, ZS, CY, FC, JL, ZZ, LL, and XL collected the data. XY, MH, YYu, XZh, MF, YL, YP, and LW wrote the original manuscript. RL, XZo, HS, YO, DX, JX, JZ, HL, HQ, XF, and CH summarized all data. ZY, SY, DZ, and YS designed the study, and revised the final manuscript. All authors contributed to the article and approved the submitted version.

## Conflict of Interest

The authors declare that the research was conducted in the absence of any commercial or financial relationships that could be construed as a potential conflict of interest.

## Abbreviations

APTT: activated partial thromboplastin time
COVID-19: coronavirus disease 2019
ARDS: severe acute respiratory distress syndrome
ECMO: extracorporeal membrane oxygenation
ELSO: Extracorporeal Life Support Organization
ICU: intensive care unit
IQR: interquartile range.
