# Supplementary material for: Extracorporeal Membrane Oxygenation for SARS-CoV-2 Acute Respiratory Distress Syndrome: A Retrospective Study From Hubei, China
Source: Front Med (Lausanne). 2021 Jan 12;7:611460. doi: 10.3389/fmed.2020.611460 (PMC7835137; doi:10.3389/fmed.2020.611460)
Supplement: Supplementary file 1 [file Table_1.pdf]

Supplementary Table 1. Analgesics, sedatives, and paralytics used during the treatment with ECMO in 73 ARDS patients with COVID-19

Medications	All patients (n = 73)
Analgesics	
Fentanyl	12 (16.4%)
Sulfentanyl	32 (43.8%)
Remifentanil	45 (61.6%)
Morphine	10 (13.7%)
Dolantin	1 (1.4%)
Butorphanol	26 (35.6%)
Nalbuphine	2 (2.7%)
Sedatives	
Midazolam	70 (95.9%)
Propofol	37 (50.7%)
Dexmedetomidine	42 (57.5%)
Promethazine	2 (2.7%)
Paralytics	
Vecuronium	4 (5.5%)
Rocuronium	27 (37.0%)
Atracuronium	25 (34.2%)
Cisatracuronium	11 (15.1%)

Abbreviations: ECMO, extracorporeal membrane oxygenation; ARDS, acute respiratory distress syndrome; COVID-19, coronavirus disease 2019.
